# Low-cost automated call box system to conduct playback experiments for wildlife research and management

**DOI:** 10.1016/j.ohx.2023.e00418

**Published:** 2023-04-08

**Authors:** Vianney J. Cupiche-Herrera, Geovanni I. Balan-Medina, José D. Cú-Vizcarra, Alexis A. Mora-Roche, Jeisson Rodriguez-Valenzuela, Brian McLaren

**Affiliations:** a Lakehead University, Faculty of Natural Resources Management, Canada; b the Intercultural Mayan University of Quintana Roo, Department of Sustainable Development, Mexico; c Institute of Ecology, A. C., Mexico; d Georgian College, Canada; e AGROSAVIA (Obonuco Research Center), Colombia

**Keywords:** Acoustic cues, Animal behavior, Automated Call Box system, Social cues, Playback experiment

## Abstract

Acoustic cues (e.g., vocalizations or ‘calls’ in wildlife) may effectively change social behavioral patterns in certain species. There are very few published papers on how to create a device at a low cost that can automatically reproduce acoustic cues to carry on wildlife playback experiments or management. We developed the Automated Call Box (ACB) based on an open-source Arduino-compatible microcontroller that attaches to a speaker system and costs less than the most common game caller used by researchers. The unit helps test the influence of social information on habitat choices and has other management applications. We field-tested the ACB to assess the effect of conspecific vocalizations cues on the habitat settlement and to increase the detectability of songbird species. We describe in detail the costs of the materials to build this type of system as a reference for further research. Our ACB allows researchers to conduct experiments on wildlife in inclement weather and complex environments, even in remote locations, with minimum or no necessity for the researcher to be present to reset the equipment.

## Graphical abstract

**Figure:**
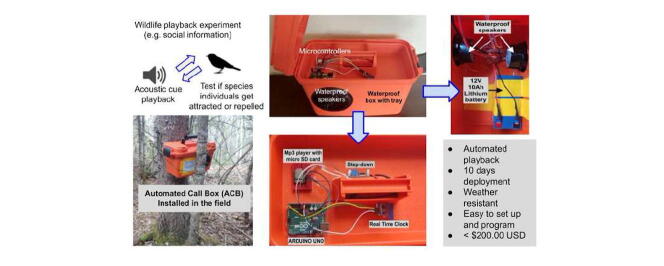

**Specifications table**

**Table:** 

Hardware name	Automated Call Box (ACB)
Subject area	• Conservation biology, environmental, educational tools
Hardware type	• Automated playback system
Closest commercial analog	“FOXPRO game caller.”
Open source license	CC-BY Attribution 4.0 international
Cost of hardware	$170.21 USD per unit
Source file repository	http://doi.org/10.17632/mwchxw4hnp.2

## 1 Hardware in context

Social interactions with other organisms may provide reliable information about the availability of a particular resource. This can permit individuals to quickly assess potential breeding sites with less energy than for a direct assessment (the social information hypothesis) [1], [2], [3]. Since vocalization is the most frequent communication in vertebrates, learning about the interactive response to acoustic cues has helped researchers understand the relationship between habitat selection and social information [4], [5].

There is evidence that acoustic signals (e.g., vocalizations or ‘calls’ in birds, frogs, and crickets) may effectively change the social behavioral patterns in certain species [6], [7], [8]. Calls can alert of a threat of predation [9], an option for a more effective search for a food source [7], opportunities for mating [10], and even a long-term suitable habitat [11]. Accordingly, many researchers use automated vocal stimulation to study species' responses to different acoustic cues (e.g., [12], [13], [14], [15]).

Most published studies briefly explain the playback protocol in their methods and scarcely describe the speaker system used. In the best cases, the studies just mention speakers encased in waterproof boxes or the brand of the game caller, but there is not enough detail to replicate the device (e.g., [12], [13], [16]). The most common alternative used in studies for animal acoustic cues is a commercial game call unit such as FOXPRO [16], [17], [18], [19], [20]. Game callers and the necessary attachments cost more than 200.00 USD per unit, making the devices less affordable for students, researchers, and conservationists who may implement more replicates than hunters are purchasing. Another disadvantage is that most of the studies using game callers do not describe how to automate the devices (without the use of remote control), and there are no instructions available (e.g. [16], [17], [18], [19]).

Few published methods exist for constructing devices designed to broadcast sound with accessible components for use in the field, requiring researchers and managers to spend much time and expense designing, building, and testing their systems [21]. In one of the methods published, most components are currently obsolete, or there are better and cheaper versions with more resistance to weather conditions (e.g. [21]). In other cases, a motion sensor typically triggers the devices, so the systems only play a sound when an animal encounters them (e.g. [2e.g. [22], [23], [24]). The broadcast by motor sensor limits functionality to test certain questions related to the social information hypothesis, such as habitat selection (for breeding, winter, or stopover habitats), mating choices, and/or triggering fright responses. To study these questions, a researcher must repeatedly reproduce the acoustic cue in the habitat to test if individuals are attracted or repelled to settle in the area due to the artificial cue.

Here, we describe an Automated Call Box system (ACB) that comes at a lower cost than game callers. We designed the ACB to be adaptable to almost any environment and weather conditions; it can broadcast acoustic cues in wildlife playback experiments in remote areas. The ACB can play calls for an extended period and assess the influence of artificial cues on species settling in determined habitats. The attraction method technique could be a relatively quick, cheap, and easy way to directly facilitate the presence or increase the density of a species [24]. By using broadcast systems of this type, we can test hypotheses about social information use and stimulate endangered species with limited ranges to expand their distributions [3], [16], [25]. In addition, such systems improve the success of translocations (see some experiments discussed in [28]); and help migrants find stopover sites in disturbed landscapes (e.g., [20], [26]). The use of the sounds could help discourage settlement by nuisance animals; also, the ACB could serve as an acoustic deterrent to prevent casualties in wind farms, roadways, or electrical transmission lines, where automatization is one of the essential requirements [15].

The ACB will provide researchers, students, and managers with a helpful alternative made with accessible and low-cost materials that will allow them to evaluate the behavioral dynamics, interaction, and habitat preferences of the species under study. The advantages of open-source Arduino boards and other microcontroller devices will reduce the need for a researcher to manage the equipment in the field and consequently reduce field expenses.

Here we provide a technical description of the device and a supporting practical guide for building and programming the ACB. We describe the successful implementation of the ACB in a playback experiment to test social information influence on the habitat settlement of a migratory songbird in the boreal forest of Northwestern Ontario in Canada. We briefly describe the results of the experiment and the performance of the ACB, such as battery durability and challenges encountered limitations, and ways to improve the system.

## 2 Hardware description

We designed the ACB with Arduino because it uses free software and is feasible to program with any protocol. The Arduino is the board that controls the rest of the ACB system’s elements using a programming code (https://doi.org/10.17632/mwchxw4hnp.2). The other boards are the RTC (Real Time Clock), which controls the time, and the DfplayerMini (Mp3 player), which plays the Mp3 file from the microSD memory card. The 12 V 10Ah lithium battery powers the system providing flexibility and extended life to the ACB. The sound comes out of a pair of marine-grade speakers, and everything is encased in a marine-grade box (water and shock resistant), protecting all the components from inclement weather.

The RTC is the ideal solution when developing systems requiring accurate timekeeping. Although other microcontrollers have internal counters, they are less accurate than a dedicated RTC. This microcontroller uses its own battery; it keeps working even when the other boards are asleep.

The DfplayerMini module is a small Mp3 audio player with an integrated audio amplifier that allows loading the playback files directly onto a micro SD memory card. It is a serial communication device using the Tx/Rx pins to connect to other microcontrollers, such as in the Arduino family. The Mp3 player turns on at the programmed time and plays the sound with the help of the speakers and SD card (the Mp3 files previously stored in the SD card).

We attached marine-grade speakers to each side of the box for a 360° audio performance. However, the design can change and adapt to different necessities. Both speakers can be attached to the same side of the box, or a cheaper system can use a single speaker. We chose marine-grade speakers to prevent failures from weather conditions and to achieve a highly weather-resistant system.

The advantages of our ACB over other proposed devices and commercial game callers commonly used by researchers are:

• It is a low-cost device with lower power consumption.
• Thanks to the open source and high availability of materials for its construction, the ACB is accessible to researchers, students, and managers.
• Components are up-to-date.
• It is relatively easy to set and program.
• It is completely automated.
• It has a longer broadcast period due to extended battery life.
• It is weather and shock resistant due to the use of a marine-grade box and speakers.
• It is easy to transport because a single box encases all components.
• It allows programmable timing of calls with high precision due to the RTC component.

We acknowledge our ACB also has some disadvantages because of the lack of video or audio recording capability. It has limits to observing particular patterns of behavioral responses. With our design, the researcher can only detect an indirect observable outcome, like habitat selection, breeding habitat selection, mate choices, or unoccupied habitat (when an acoustic cue may have deterred the species from settling in or occupying the habitat).

### Design files

**Fig. 1:**
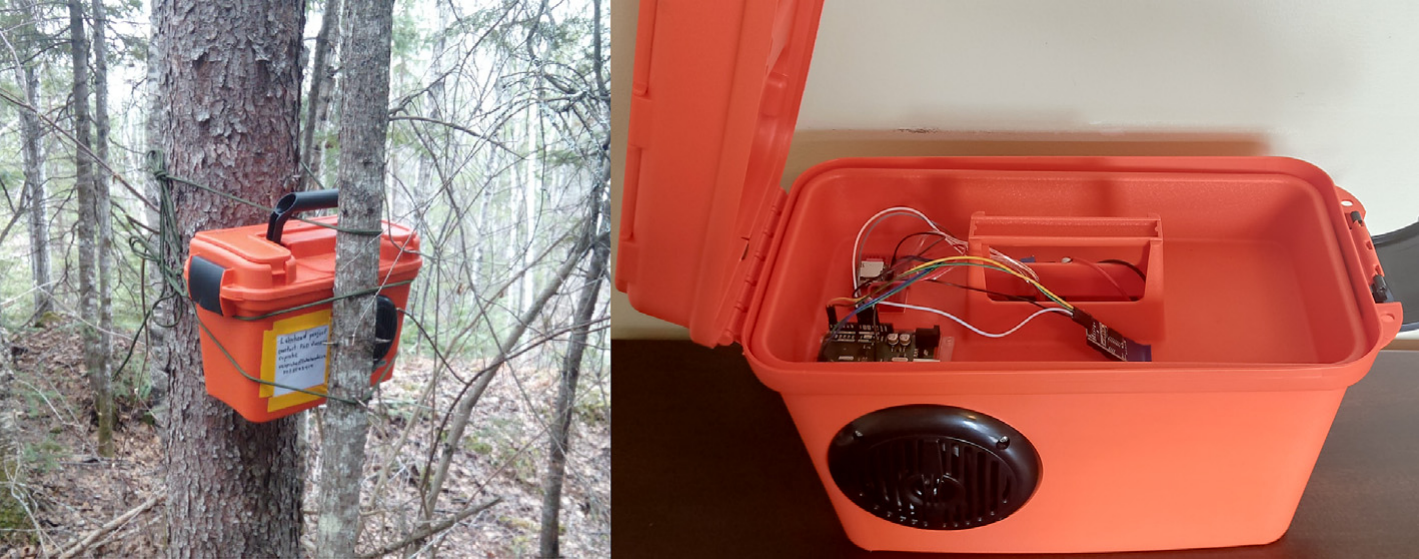
Left image: ACB (Automated call box) installed in the field. Right image: an overview of the system encased in the waterproof box. The photos show how we set up the ACB in the field during the experimental test and the overview of the system encased in the waterproof box. We placed the microcontrollers in the box tray. We attached the speakers to the front and back of the box (the battery is inside the bo; see Fig. 3).

**Fig. 2:**
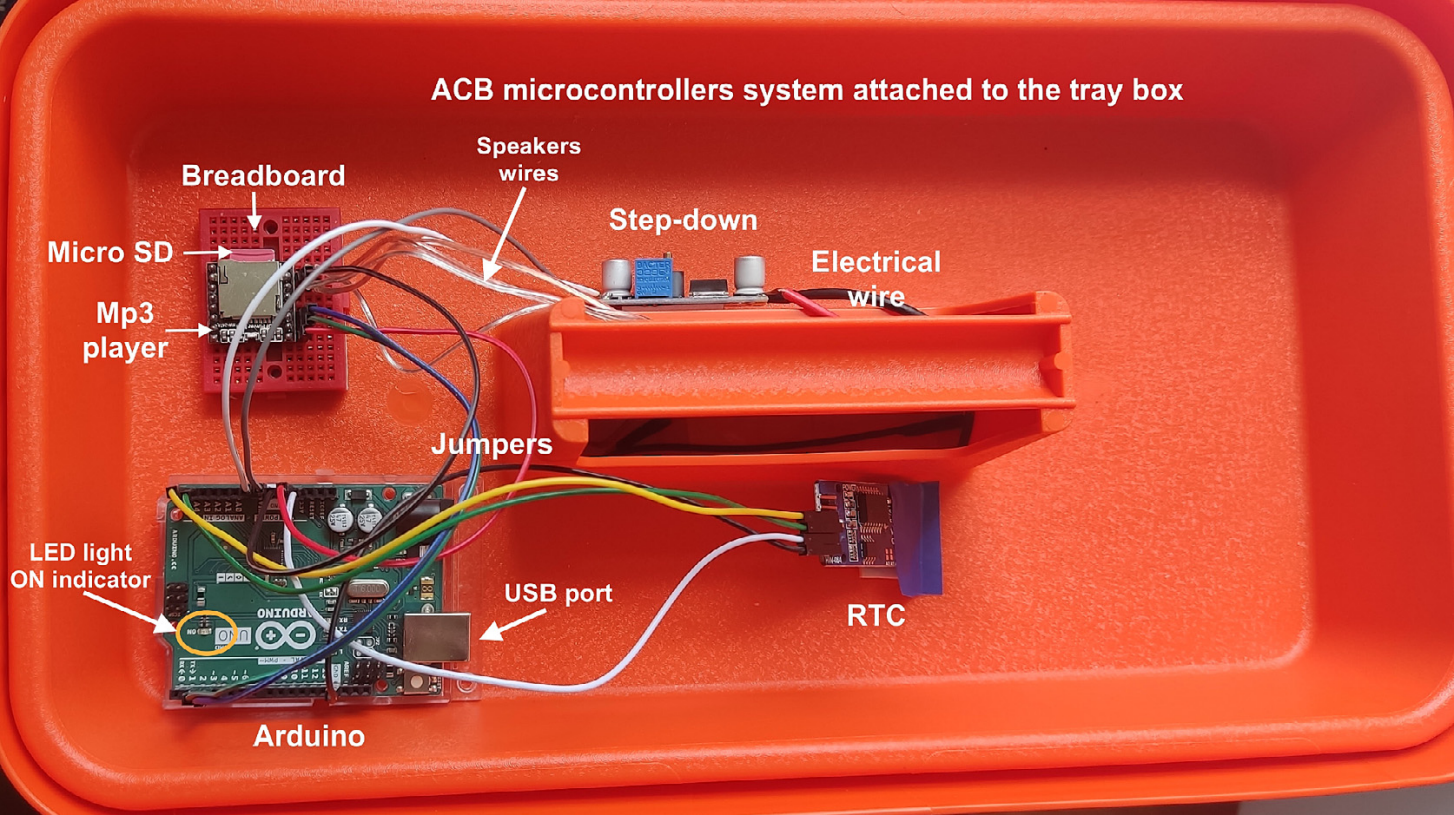
Overview of the ACB system: the Arduino (to set the automated playback program), the mini breadboard (to connect the MP3 module to Arduino and speakers), the micro SD card (to store the playback songs), the step-down (to convert voltage from 20 to 12 V to 5 V), and the RTC (Real Time Clock to set the time for the automated system). The image shows how we arranged all boards to achieve good system performance.

**Fig. 3:**
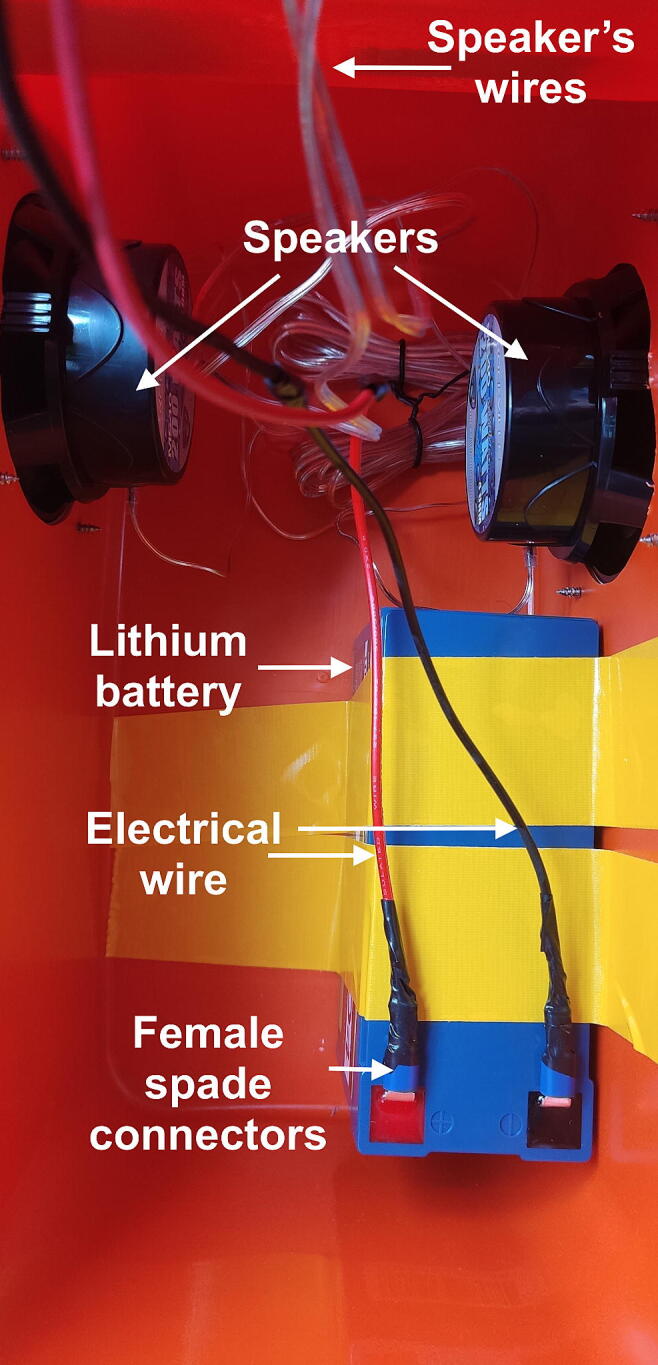
Overview of the battery and speakers inside the box. The image shows how the speakers and the battery were attached to the box. We attached the speakers to the box's front and back for a 360° audio performance. We fixed the battery to the box's interior to protect it from inclement weather and to avoid its movement.

**Fig. 4:**
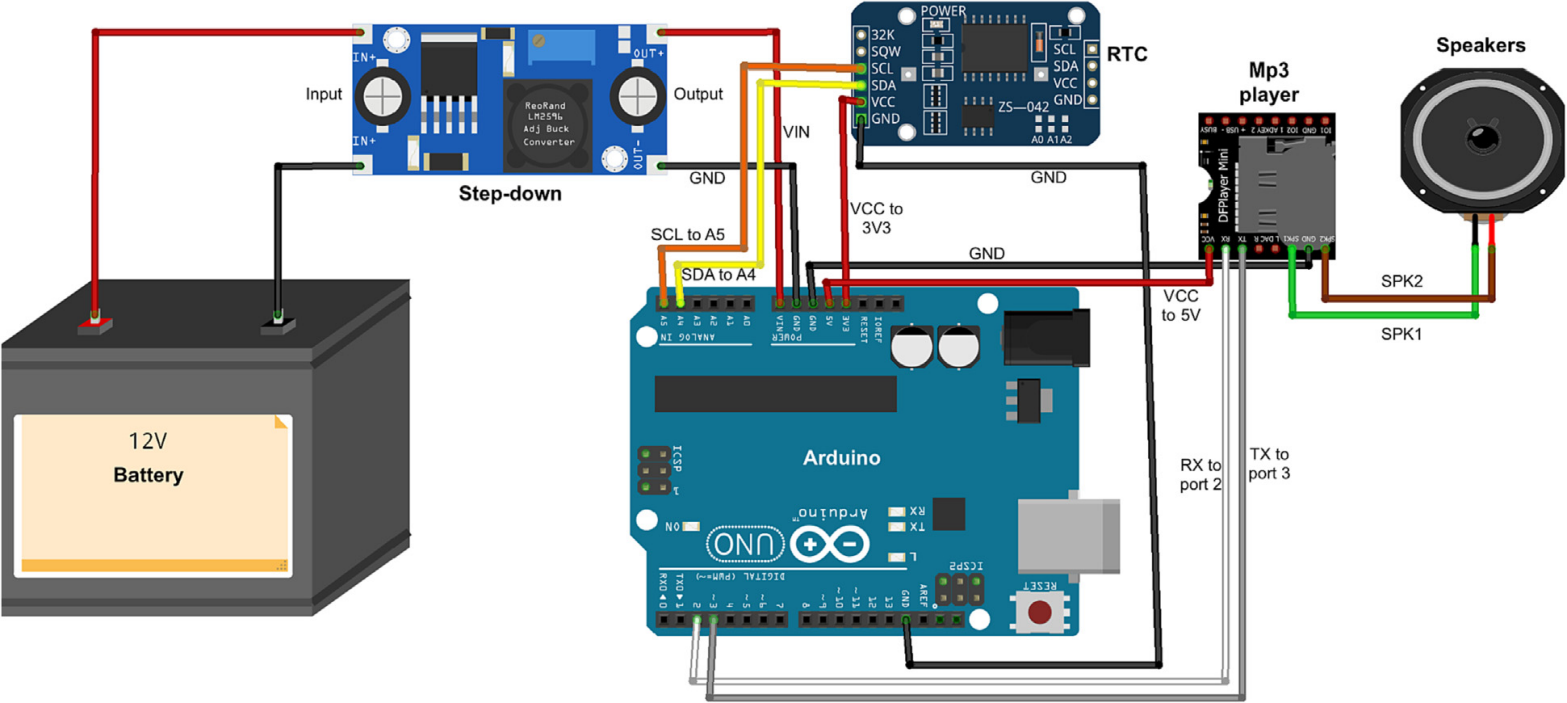
ACB cable set up. The color of the wires is just a guide; they can be different according to availability. The orange wire connects the SCL (RTC board) and the A5 (Arduino) pins. The yellow wire connects the SDA (RTC board) and the A4 (Arduino) pins. The gray wire connects the TX (Mp3 player) and the −3 (Arduino) pins. The white wire connects the RX (Mp3 player) and the 2 (Arduino) pins. Red and black wires are standard connecting the positive and negative (ground) pins. (For interpretation of the references to color in this figure legend, the reader is referred to the web version of this article.)

The design files consist of the overall design and guide of cable setup to assemble the necessary components to build the ACB (Fig. 1, Fig. 2, Fig. 3, Fig. 4, and Table 1).

**Table 1:** ACB design files.

**Design file name**	**File type**	**Open source license**	**Location of the file**
Hardware	JPG, Fig. 1, Fig. 2, Fig. 3, Fig. 4	CC-BY Attribution 4.0 International	“Available with the article.” http://doi.org/10.17632/mwchxw4hnp.2
Program sketch	Arduino file	CC-BY Attribution 4.0 International	https://github.com/Vcupiche/ACB http://doi.org/10.17632/mwchxw4hnp.2

## 3 Design files summary

Table 1 contains the list of design files used in the ACB construction. The ACB includes a custom-made Arduino-based circuit and speaker unit encased in a weatherproof plastic box designed to be used outdoors and automated (Fig. 1).

### Bill of materials

We selected the microcontrollers based on their low battery consumption and cost (see Table 2 for specifications and expenses). The cost of ACB is source dependent. Most of the prices in the bill summary are from Amazon.com; major costs include the battery, speakers, enclosure, and Arduino board. Other boards, micro SD cards, and wires incurred minor costs; we could buy these items in bulk (various pieces) for a lower price. The total unit cost, which includes cables and equipment required to manufacture (e.g., soldering iron), is less than $190.00 USD per unit. Non-original brand purchase of materials could reduce cost but can lead to loss of quality and durability.

**Table 2:** Bill of materials necessary for building an ACB in USD.

**Designator**	**Component**	**Number**	**Cost per unit -currency**	**Total cost -** **currency**	**Source of materials**	**Material type**
Speakers	Pyle, Marine-grade speakers 4″ 100 W 4 O waterproof	1	21.20	21.20	Amazon	electronic and metal
Lithium battery	12 V 10Ah LiFePO4 deep cycle battery	1	70.00	70.00	Amazon	lithium
Step-down	Adjustable step-down module LM296 DC-DC buck converter 3.0–40 V to 1.5–35 V	1	2.00	2.00	Amazon	other
Arduino	Arduino Uno SMD REV3	1	28.50	28.50	Amazon	electronic
Mp3 Player	DfMini Player Mp3 Board YX5200 Module Support Micro SD card	1	5.00	5.00	Amazon	electronic
Breadboard	170 tie-points mini breadboard for Arduino projects	1	2.50	2.50	Amazon	other
SD card	micro SD card, 8 GB (or any other capacity as needed)	1	6.00	6.00	Amazon	memory
RTC (real-time clock)	DS3231SN IIC RTC sensor high precision timer alarm clock breakout memory board	1	5.00	5.00	Amazon	electronic
Clock battery	CR2031 clock battery	1	2.15	2.15	Amazon	lithium
Box	Flambeau, Marine dry box 14″	1	25.86	25.86	Amazon	plastic
Jumper wires	Multicolored jumper wires, male to female, male to male, and female to female 15–20 cm	10	0.20	2.00	Amazon	cooper and plastic
Connectors	Female spade connector 16–14 gauge, nylon insulated	2	0.25	0.50	Amazon	metal and plastic
	**total per unit**			170.21		

## 4 Bill of materials summary

We present the list of materials and costs in Table 2. The bill list does not include the cost of items such as a digital multimeter, soldiering iron, tapes, and other materials necessary to construct the ACBs. These costs can be highly variable. We list the required tools and other materials with the instructions on building.

Most items (i.e., boards, clock battery, micro SD) are available in bulk, in the amount of five to six pieces. Buying in bulk reduces the cost of each ACB. There are other options to buy the items at a lower price, like at Aliexpress (https://www.aliexpress.com), but they have longer shipping times and sometimes higher import fees.

## 5 Building instructions

**Fig. 5:**
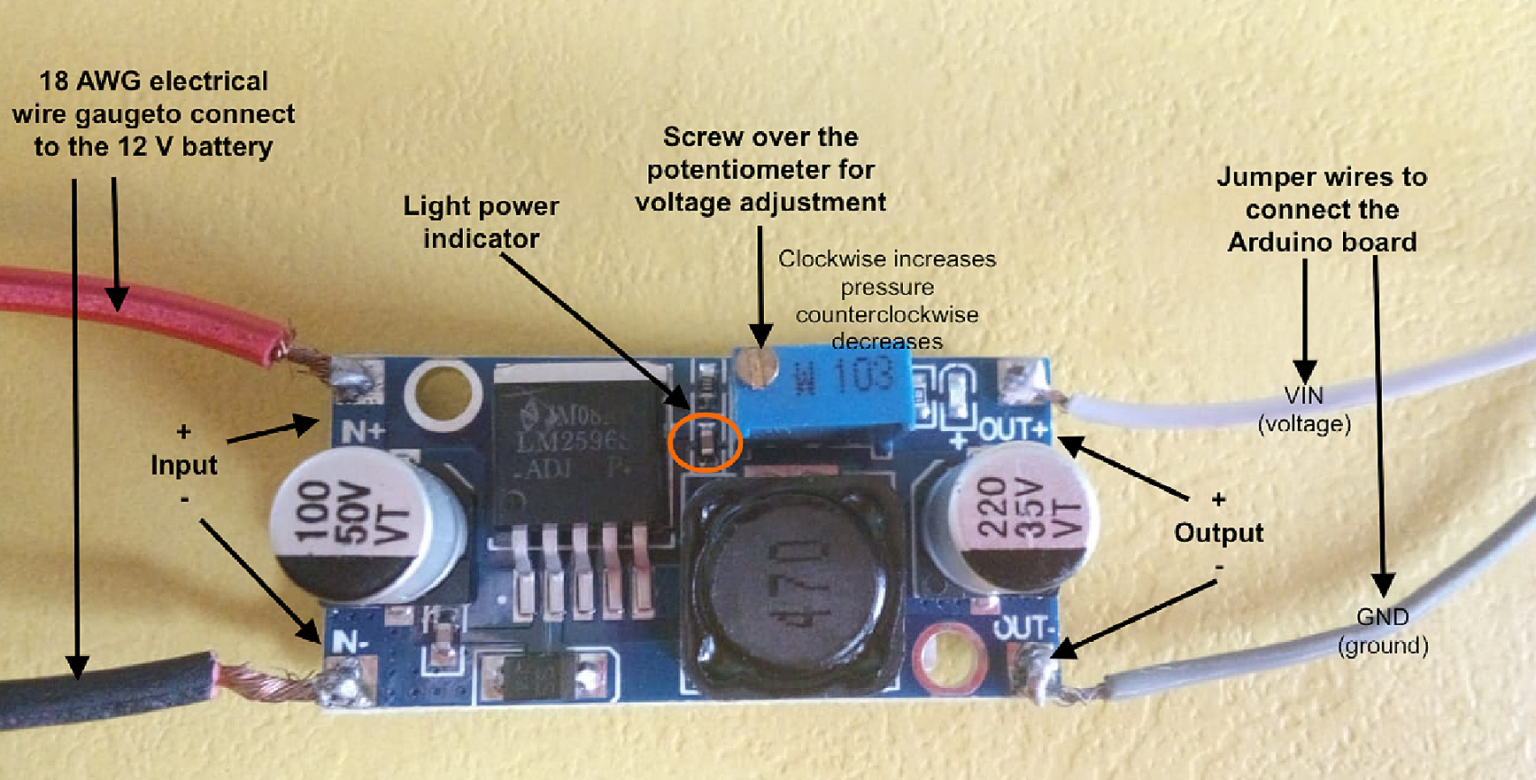
Step-down closer view. Jumper and electrical wires soldered to the step-down. The image shows the screw that helps to calibrate the step-down.

**Fig. 6:**
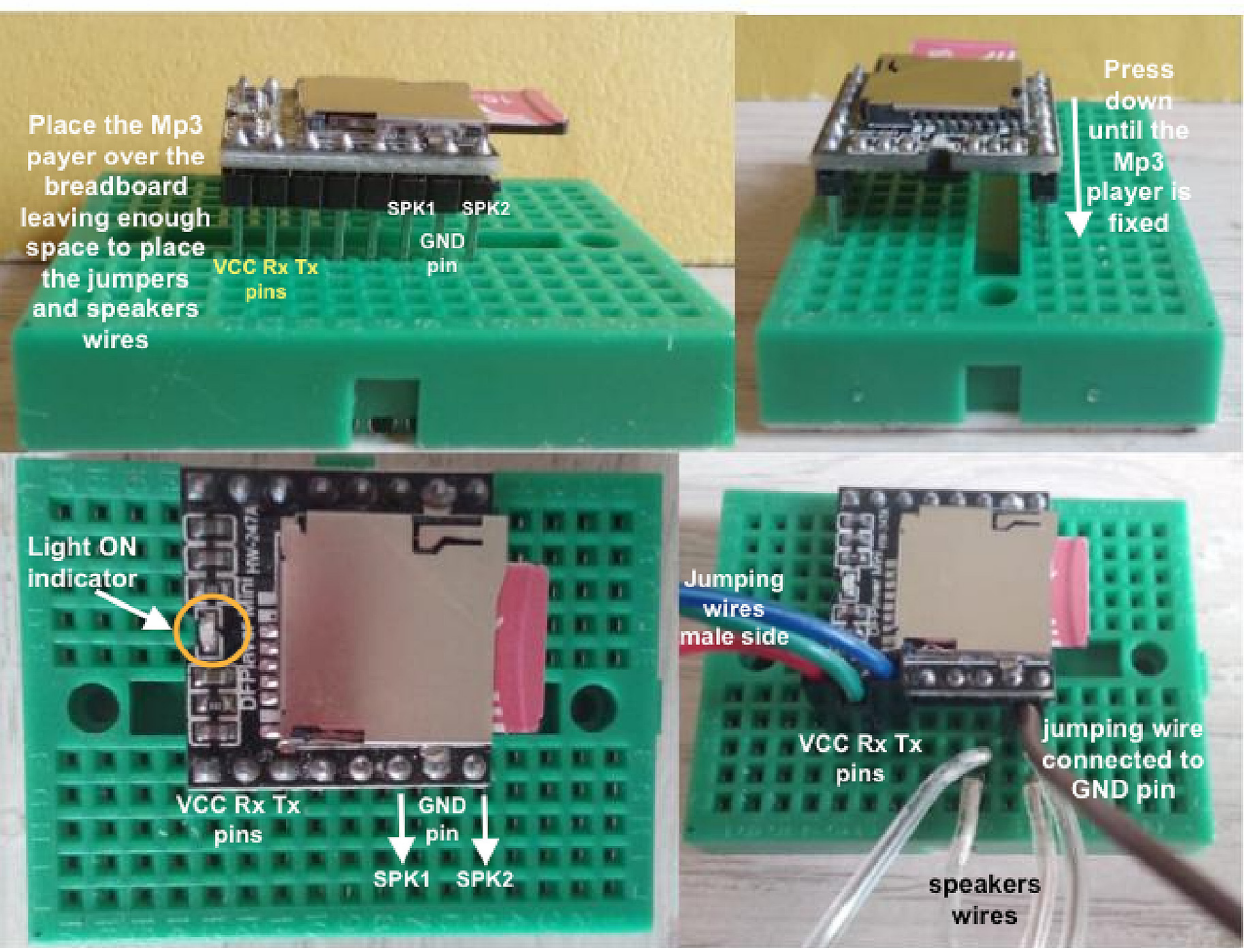
Closer view of the Mp3 player assembled on the breadboard. The image shows how we set the Mp3 player on the breadboard. Also, the image shows the SD card inside the Mp3 player. The image in the bottom right also shows the jumper wires: red for the VCC pin, green for the Rx pin, blue for the Tx pin, brown for the GND pin (colors may change, we used male-to-male wires), and the speaker's wires (transparent ones) connected to the Mp3 player’s SPK1 and SPK2 pins through the breadboard. (For interpretation of the references to color in this figure legend, the reader is referred to the web version of this article.)

**Fig. 7:**
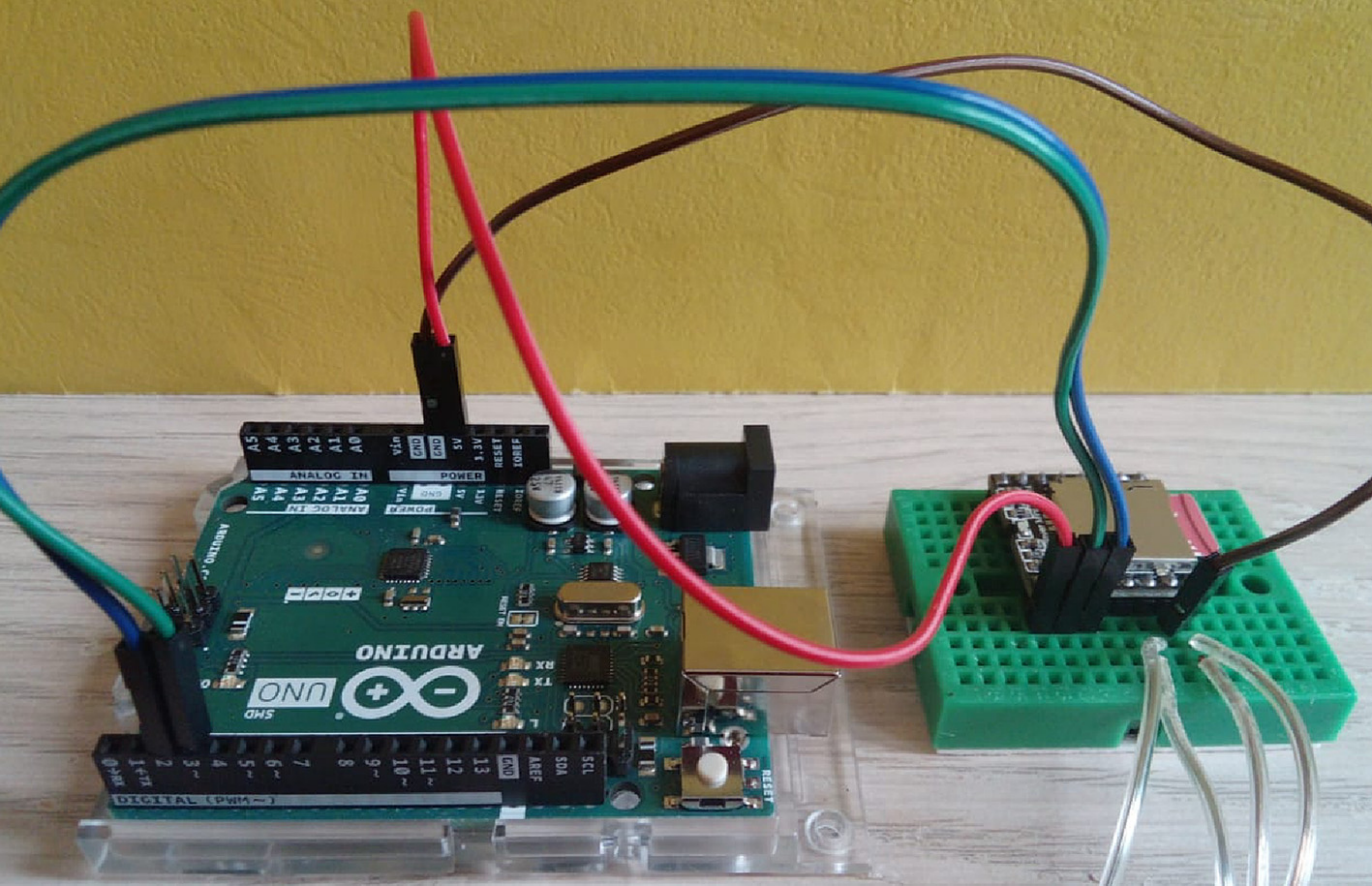
Closer view of the Arduino board and the Mp3 player connection (wire color is up to the user selection). The image shows how we attached the jumper wires (male-to-male wires). The brown wire connects the Mp3 player and the Arduino’s GND pins. The red wire connects the Mp3 player’s VCC pin to the Arduino’s 5 V pin. The green and blue wires connect the Mp3 player’s TX and RX pins to the Arduino’s −3 and 2 pins (respectively). (For interpretation of the references to color in this figure legend, the reader is referred to the web version of this article.)

**Fig. 8:**
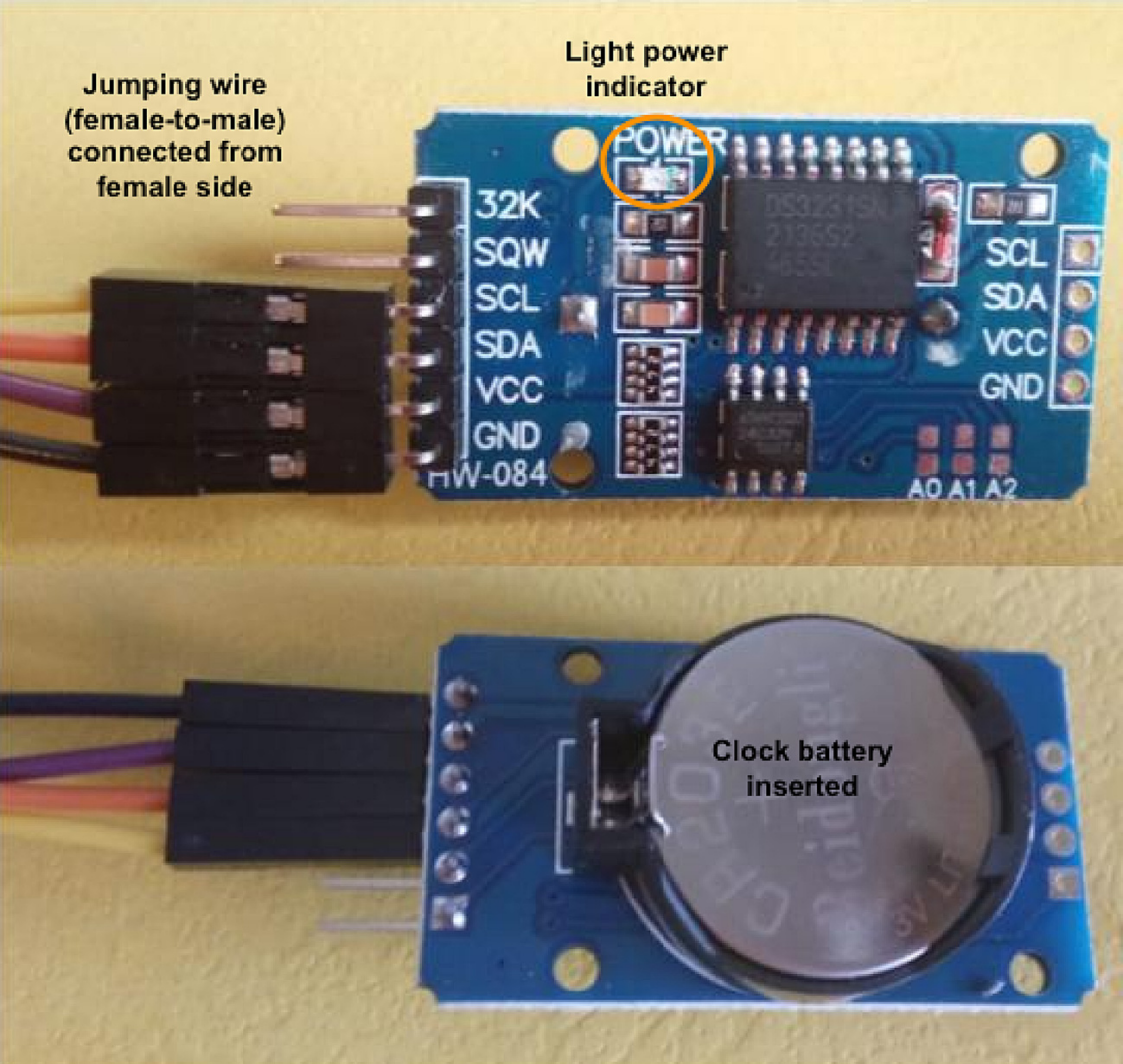
Closer view of the RTC (Real time clock) with its battery and jumper wires connected (female side).

**Fig. 9:**
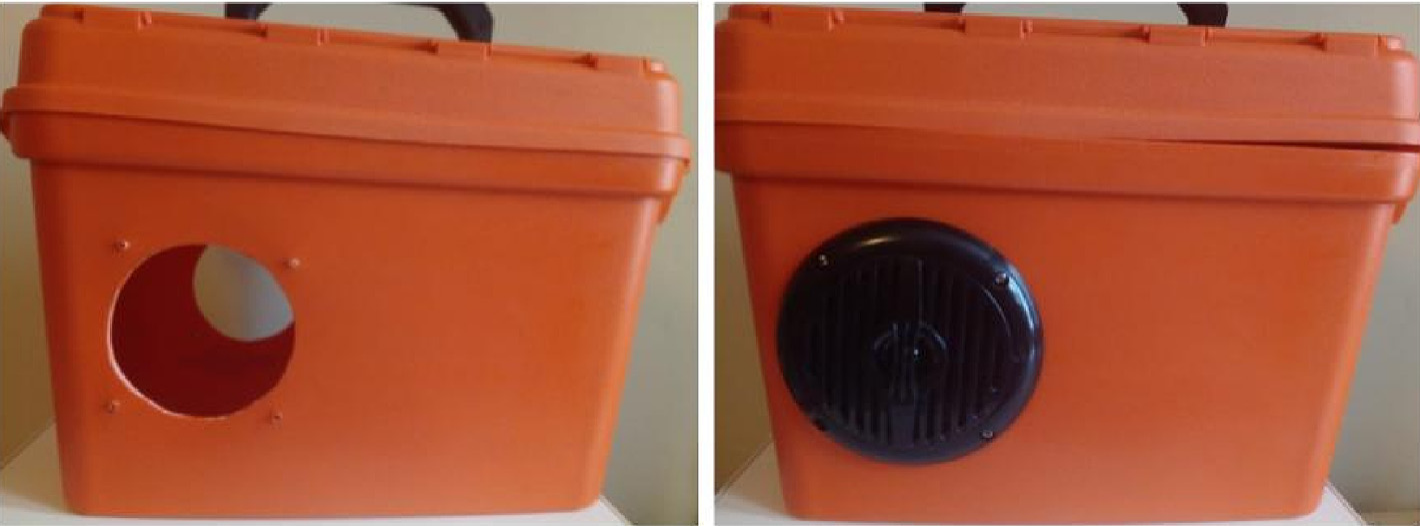
Speaker installation on the box. The left image shows the holes and perforations made to the box to attach the speakers; we followed the speaker's instructions to draw the circle on the box and used a cutter to make the holes. The right image shows the speaker attached to the box by pressure, and we fixed them with the help of stainless screws to avoid movements or accidental detachment.

We show the overall design and guide the assembly of the components in Fig. 1, Fig. 2, Fig. 3, Fig. 4; a closer view of certain components and steps required to assemble the system are in Fig. 5, Fig. 6, Fig. 7, Fig. 8, Fig. 9. In conjunction with the Arduino, the RTC allows us to keep the system asleep until calls start. The RTC's battery enables it to work independently and keep the time running. The Mp3 player can read mp3 files sorted on a micro SD card and reproduce audio through the speakers. Finally, the marine-grade box greatly resists adverse weather conditions (rain, dust, wind) and gives flexibility in installation and easy transportation in the field.

The construction of the ACB requires some necessary tools and materials, which are the following:

• Digital multimeter.
• Soldering iron.
• Battery charger with pliers 20 V (one is enough to recharge several batteries).
• Wire cutters/strippers.
• Solder wire WT50g.
• Vinyl electrical insulating tape.
• AWG electrical wire gauge red and black 5–12 V DC.
• Pliers.
• Phillips head screwdriver.
• Stainless screws (eight).
• Double-sided adhesive pads.
• General-purpose resistant tape.

Before assembling everything, we need to calibrate the step-down to 5 V using the digital multimeter because the Arduino is more stable with this voltage:

1. Turn on the multimeter and set it up at 20 V.
2. Connect the multimeter's positive (red) and negative (black) wires into it.
3. Connect the other side of the cables to the step-down output, with red cable in the positive (+) and black in the negative (-).
4. Connect the step-down input side to a battery or charger (20 V); the red wire for the positive (+) and the black for the negative (-).
5. Using the Phillips head screwdriver, twist the screw over the step-down (Fig. 5) until the voltage on the multimeter screen appears at 5 V.

### Boards assembly

For assembling the microcontrollers, it is necessary to have a certain type of jumper wires depending on the type of pins:

• For attaching the step-down to the Arduino, we used any jumper with a male side (we cut the other side for soldering).
• For the Mp3 player is necessary to use male-to-male wires.
• For the RTC, it is necessary to use female-to-male wires. The female side attaches to the RTC, and the male side attaches to the Arduino board.

Once we calibrated the step-down and have the necessary components, we can assemble all the boards:

1. Attach four wires to the step-down: two jumper wires to the output side and two strips of electrical wire gauge (red for + and black for -) to the input side. It is necessary to use the soldering iron to attach the wires to the step-down (Fig. 5).
2. On the other side of the electrical wires, it is necessary to attach female connectors using the vinyl electrical insulating tape (Fig. 3). The female connectors will help to easily connect and disconnect the 12 V battery to the system.
3. Attach the step-down to the box tray using double-sided tape; the best place is next to the tray handle (Fig. 2).
4. Fix the Arduino board to the box tray through the tape included in the microcontroller base. Locate the Arduino in a manner that leaves free space for the USB port.
5. Connect the step-down to the Arduino using the jumper wires from the output side (male side). Connect the positive (+) output wire to the VIN (voltage input) entrance and the negative (-) output wire to GRD (ground entrance); see Fig. 4, Fig. 5.
6. Place the Mp3 player on the breadboard and fix the breadboard to the tray; the best place to locate it is beside the step-down (Fig. 2). The breadboard helps to connect the Arduino and speakers to the Mp3 player (Fig. 4, Fig. 6, Fig. 7).
7. The Arduino powers the Mp3 player board. Connect the boards using the VCC pin from the Mp3 player to the Arduino’s 5 V pin, and the GND pin should be linked (Fig. 4, Fig. 7).
8. The Mp3 player board receives the signal from Arduino connecting the Rx and Tx pins (from the Mp3 player) to the 2 and 3 pins (from the Arduino); see Fig. 4, Fig. 6.
9. Insert the clock battery into the RTC and connect the jumper wires (female side, Fig. 8) to be attached to the Arduino board. Fix the RTC to the box tray using vinyl tape and locate it on the opposite side of the tray handle (Fig. 2).
10. Connect the RTC using the SCL and SDA pins to the A5 and A4 from the Arduino. Connect the RTC at the VCC and GND pins to the 3 V and GND entrances from the Arduino board (Fig. 4).

### Speaker and 12 V battery installation

Attach the speakers to the front and back of the box by making a hole following the speaker's instructions. Then insert the speakers into the hole and fix them with stainless screws to finish the installation (Fig. 3, Fig. 9). Connect the speakers to the Mp3 player’s SPK1 and SPK2 pins by using the breadboard (Fig. 4, Fig. 6).

Attach the 12 V battery inside the box in a corner, giving space to the speakers (Fig. 3). Use double-sided adhesive pads and general-purpose-resistant tape to fix the battery; it will avoid movements and connectivity failure. Connect the battery to the system through the electrical wires from the step-down using female connectors (Fig. 3, Fig. 4).

## 6 Operation instructions

We used the ARDUINO IDE (Integrated Development Environment) because is free and open-source software for programming the ACB. This software is easy to download through the official Arduino webpage (https://www.arduino.cc). We must install and open Arduino; we developed a custom sketch (programming code) that is easy to set and adapt to different necessities; it is available at http://doi.org/10.17632/mwchxw4hnp.2. Before running the code, it is necessary to install two libraries: RTClib and DFROBOTdfplayermini; which provides the sketch the ability to keep the system asleep until the set time; when the system wakes up, it plays the Mp3 and returns to sleep.

Using a printer USB cable (A male to B male), we will connect the Arduino board to a computer to upload the sketch. It is essential to avoid plugging in the Arduino board and the system to the 12 V battery and the computer simultaneously; an additional power source can burn out the entire system. Before coding, it is essential to ensure the correct installation of all the boards; when we plug in the battery to the system or the Arduino to the computer, all the LED boards must turn on (Fig. 5, Fig. 6, Fig. 7 show the location of the LEDs lights). If the lights are off, we must verify the wires connections.

We will program the RTC using the time on the computer through the Arduino sketch. To ensure time accuracy, we must verify the time on the computer. If the Arduino monitor gives a warning indicator about the RTC or Mp3 player, we must ensure the wires are well-connected and appropriately placed. We developed the starting time, length, and frequency programmed in the sketch for diurnal songbirds, but we can adapt the code according to the research needs (changing the time and frequency).

For more accessible coding, we should name the Mp3 files using numbers or letters (1, 2, 3, and so on, or A, B, C). We can add more Mp3 files and program the ACB to call each file at different times, but we have to arrange the files in the sketch in the order we stored them on the SD card. The ability of the ACB to program play times is valuable in setting the acoustic cue at the most suitable time for the target species. We can program the ACB in a way such that the broadcast does not become routine, avoiding the habituation of the species. We provide more details within the commented code and the code programming description guide (available at http://doi.org/10.17632/mwchxw4hnp.2 and https://github.com/Vcupiche/ACB).

We can disconnect the device from the computer and plug it into the 12 V battery when we finish setting the program. The ACB will stay completely off if we do not plug it into the 12 V battery. When we require to set the ACB in the field, we only need to plug the electrical wires from the output step-down to the 12 V battery (Fig. 3), and the device will be ready to work.

## 7 Validation and characterization

We constructed six ACBs to conduct a playback experiment to test the social information influence on songbird species habitat settlement in Northwestern Ontario, Canada. Before carrying out the experiment, we tested the system under controlled (indoors) and uncontrolled conditions (forested areas within and surrounding Thunder Bay city in Ontario, Canada) in May 2022. We used the flexibility of the ACB components to program some songbird songs and calls to test the system; we implemented species vocalizations such as White-throated Sparrow (*Zonothrichia albicollis*), American Redstart (*Setophaga ruticilla)*, Ovenbird (*Seiurus auroapilla*), Black-capped Chickadee (*Poecile atricapillus*), and Magnolia warbler (*Setophaga magnolia*). We programmed the ACB to broadcast the tracks from 6:00 am to 1:00 pm every 15 min; each track lasted at least 15 min.

All the systems worked as expected; the volume was sufficiently loud under controlled conditions, and the battery lasted 11 days. Under uncontrolled conditions, the performance of the system slightly decreased; we could hear the recordings at a distance of greater than 50 m even with surrounding sounds (e.g., traffic, roads, people, other animal vocalizations, wind) and high tree density. The battery lasted only 10 days due to cold temperatures (0–2 °C). Individuals of all species used in the experiment approached the ACB installation site when the system broadcasted the playbacks.

### Social information experiment

We used the migratory songbird Canada warbler (*Cardellina canadensis*) as the study species. The Canada warbler (CAWA) is a species at risk [27] that could have evolved to respond to conspecific cues due to its short breeding season, which leaves limited time for individuals to assess habitat quality and search for mates [28]. There is evidence of the influence of CAWA habitat use by conspecific attraction in harvested forests [28], [29]. However, there is a knowledge gap on the influence of conspecific attraction on CAWA habitat selection.

We conducted a survey during the early spring migration season (late May to mid-June) of 2022 when the migratory species searched for breeding habitats [30]. We selected each ACB installation site based on a previous bird survey in 2021; we used sites where we did not detect any CAWA (vacant sites) during the 2021 breeding season to test if males of the species are attracted to settling there. We verified in each location the absence of CAWA before placing the ACBs on each vacant site. We installed and programmed the ACBs to run for seven days; we recharged them after this period and re-installed them in different areas until we completed 18 locations on three landscapes (six per landscape). Also, we surveyed another set of vacant sites (10 per landscape) using a silent point count protocol as the control treatment. We used the following sequence of tracks in each ACB: 15 min of continuous songs and calls of the CAWA and 15 min of silence; the tracks were programmed to play from 5:00 am to 12:00 pm, the more active time for bird calls and songs.

We placed the ACB at least 1.5 m to 2.0 m from the ground by attaching it to a tree (Fig. 1). The position matches the species' preference to forage at the shrub level. We went back to each site to review the system between the eighth and ninth day of installation to have the chance to verify the system’s performance before the 10-day battery life ended (as we previously tested) and to recharge fully before re-installing in other sites. When the protocol finished, we went back two times (late June and early July) to each site and verified the species occupancy or vacancy; we considered CAWA to have been attracted if we observed or listened to the species within a 60–100 m radius of the ACB installation site.

### Main results

We successfully attracted seven CAWA males to seven different sites from among the 18 sites previously determined to be vacant. The sites where we attracted the individuals had more than 40% shrub cover and canopy height greater than 10 m with deciduous species predominance. We verified that CAWA male individuals established their territories after the 8–9 days playback period, returning two times to the same site. The sites where we did not attract males (11 sites) presented less than 40% shrub cover, the canopy height was lower than 10 m, and pines were the predominant tree species. We verified that CAWA did not occupy control vacant sites after the survey period; these sites have a gradient in shrub cover and canopy height.

The results suggest the interaction of conspecific cues and vegetation characteristics influences the attraction of CAWA individuals. Our results and observations in the field also suggest that the ACB successfully attracted the species; in all seven sites where we found CAWA, while we were visiting the locations after the playback protocol, we observed the species approached the site when the system started to broadcast the songs and calls.

The ACBs served their function during the experiment in a single season (late May-early July 2022). The timekeeping was accurate, and the sound kept its performance throughout the experiment. The devices worked well in different weather conditions, including temperatures ranging from 0 to 28 °C, heavy rain (greater than7 mm), and moderate winds (greater than20 km/h). We only had a technical failure in one ACB because the electrical wires were not well attached to the 12 V battery, but we noticed this issue before installing the system, so we were able to fix it. Also, one of the ACBs had a bear encounter; nonetheless, all the components withstood the attack. It was possible to reuse the whole system even when some elements fell into the water (we found one set of speakers submerged in a small pond).

After each use of the ACB (8–9 days deployment), we recharged the 12 V batteries even when they were not fully discharged; it took an average of 4 h per battery to fully recharge, and when the batteries completely discharged, it took 5 h to recharge. Once the experiment ended, we verified the performance again under controlled conditions. The ACBs worked similarly to the initial trial: 10–11 days of deployment, the playback broadcasted at the programmed time and had a good sound performance.

### Recommendations to ensure the best performance

Before placing the ACB in the field, we recommend testing its performance (time accuracy when broadcasting the playback, sound performance, and wire connections) in a lab or under controlled conditions. It will help to prevent any ACB failures in the field and change any detail we need in the sketch for the research protocol or fix in advance any hardware issues. When we set up the ACB in the field, we needed to bring essential tools to repair possible issues with the system, such as detached boards and disconnected wires. We recommend having available the following tools: wire cutters/strippers, vinyl electrical insulating tape, pliers, Phillips head screwdriver, double-sided adhesive pads, general-purpose resistant tape, extra clock batteries, and extra jumper wires.

To prevent non-desired curious animals from approaching the ACB, we recommend painting the box with a camouflage design or getting a box with a more discrete color. Another option could be covering the ACB with branches and leaves, trying not to block the speakers.

## 8 Conclusions

We described a low-cost, open-source hardware design for conducting fieldwork experiments in research into acoustic signals as part of learning about behavior and ecology. The Automated Call Box (ACB) is an easy-to-build and low-power consumption device, and it is helpful for many different applications in systematic experiments or management. The open-source characteristics we describe allow using several acoustic cues (i.e., environmental or anthrophony), which we can use to evaluate responses in attraction, deterrence, or observations of behavioral reactions. The findings of these evaluations are valuable in ecological and conservation fields. Finally, using this device in future studies could represent more precision, practicality, and low cost for researchers adopting this type of hardware.

The ACB has the following capabilities:

• The ACB has good reliability in deployment time.
• Once installed, the ACB deployment is automated and does not require the presence of the researcher to turn it on and off.
• The system has 10 days of deployment without recharging the 12 V battery. It takes less than 5 h to recharge each battery. Also, attaching a solar panel to the system can make the battery last longer.
• The ACB sketch allows users to change and program different audio cues, specific times, and days.

The ACB limitations are the following:

• Operation of the device in temperatures lower or above the ones tested would interfere with the deployment duration and microcontroller functionality. However, in the case of lower temperatures (below 0 °C), it is possible to add insulation material to the top of the box to protect microcontrollers and make the ACB work well.
• Mp3 files should have good sound quality for the best sound performance using the ACB. Due to the lack of an integrated amplifier, it is not possible to modulate the sound volume. Nonetheless, adding an amplifier can increase the device's volume and/or module it as needed.
• The system has limitations in use for species with ultrasound or infrasound ranges, such as bats and other mammals.
• The ACB’s current version does not allow for audio or video recording. However, the components' flexibility allows the user to find a way to attach other equipment (microphone or camera) if necessary.

## Ethics statements

The present study did not trap or extract any animal from the field, and we did not house or handle any animal during the experimental test.

## CRediT authorship contribution statement

**Vianney J. Cupiche-Herrera:** Conceptualization, Funding acquisition, Investigation, Methodology, Software, Writing – original draft, Writing – review & editing. **Geovanni I. Balan-Medina:** Methodology, Investigation, Resources. **José D. Cú-Vizcarra:** Investigation, Writing – original draft, Writing – review & editing. **Alexis A. Mora-Roche:** Software, Conceptualization, Writing – original draft, Writing – review & editing. **Jeisson Rodriguez-Valenzuela:** Writing – original draft, Writing – review & editing. **Brian McLaren:** Supervision, Writing – review & editing.

## Declaration of Competing Interest

The authors declare that they have no known competing financial interests or personal relationships that could have appeared to influence the work reported in this paper.
